# *Ganoderma zonatum* Is the Causal Agent of Basal Stem Rot in Oil Palm in Colombia

**DOI:** 10.3390/jof8030230

**Published:** 2022-02-26

**Authors:** Sandra Yulieth Castillo, María Camila Rodríguez, Luis Felipe González, León Franky Zúñiga, Yuri Adriana Mestizo, Héctor Camilo Medina, Carmenza Montoya, Anuar Morales, Hernán Mauricio Romero, Greicy Andrea Sarria

**Affiliations:** 1 Pest and Disease Program, Colombian Oil Palm Research Center—Cenipalma, Bogotá 111211, Colombia; sycastillo@cenipalma.org (S.Y.C.); lufgonzalezcon@unal.edu.co (L.F.G.); lzuniga@cenipalma.org (L.F.Z.); ymestizo@cenipalma.org (Y.A.M.); hmedina@cenipalma.org (H.C.M.); amorales@cenipalma.org (A.M.); 2 Biology and Plant Breeding Program, Colombian Oil Palm Research Center—Cenipalma, Bogotá 111211, Colombia; mcrodriguez@cenipalma.org (M.C.R.); cmontoya@cenipalma.org (C.M.); hromero@cenipalma.org (H.M.R.); 3 Department of Biology, National University of Colombia, Bogotá 111321, Colombia; 4 Cenipalma, Experimental Field Palmar de La Vizcaína, Km 132 Vía Puerto Araujo-La Lizama, Santander, Barrancabermeja 111611, Colombia

**Keywords:** *Elaeis guineensis*, pathogenicity, phylogenetic analysis

## Abstract

Basal stem rot (BSR), caused by *Ganoderma* spp., is one of the most important emerging oil palm diseases in Colombia, and is restricted to two oil palm production areas in the country. To identify the causal agent of the disease, basidiocarp of oil palms affected by BSR were used to prepare isolates, and their pathogenicity was then assessed in pre-nursery plants. Four-month-old oil palm seedlings were inoculated with rubber wood (*Hevea brasiliensis*) blocks colonized with dikaryotic mycelia of *Ganoderma*. The incidence, severity, and symptoms of the pathogen were assessed. A multiregional analysis (*ITS*, *rpb2*, and *tef1-α*) was carried out to identify the isolates; all isolates were determined to be *Ganoderma zonatum*. Phylogenetic analyses with the three regions yielded concordant phylogenetic information and supported the distinction of the isolates with high bootstrap support. Seven isolates (CPBsZN-01-29, CPBsZN-02-30, CPBsZN-03-31, CPBsZN-04-34, CPBsZN-05-35, CPBsZN-06-36, and CPBsZN-07-38) were pathogenic in oil palm, with incidences greater than 90% and a maximum severity of 34%, and the highest severity index was found in isolates CPBsZN-03-31, CPBsZN-04-34, and CPBsZN-06-36. The pathogen was recovered from inoculated oil palms in all cases. This study reveals the pathogenic association of *Ganoderma zonatum* with BSR in Colombia.

## 1. Introduction

Oil palm (*Elaeis guineensis* and interspecific hybrid OxG (*Elaeis oleifera* X *Elaeis guineensis*)) is one of the main agro-industries in Colombia, with a planted area of more than 559,000 hectares and a palm oil production of 1,528,739 tons, ranking fourth as a producer worldwide [1]. Crop health is one of the main limiting factors, and bud rot and lethal wilt are the most important oil palm diseases. However, basal stem rot (BSR), which is still restricted to two oil palm production areas of Colombia, is one of the most relevant emerging problems that affects oil, even in other countries in the world. BSR is the most important disease in Malaysia and Indonesia, which are the world’s leading producers of crude palm oil. The economic losses caused by this disease in Indonesia are estimated to be USD 256 million per year for each 1% of infections by *Ganoderma boninense* [2,3].

Although various studies on BSR have been carried out in Colombia, the causal agent of the disease is still unknown. The species *G. boninense, G. zonatum,* and *G. miniatocinctum* have been associated with BSR of oil palm in other regions of the world where this crop is grown [4,5]. Among the different *Ganoderma* species associated with oil palm production in Southeast Asia, *G. boninense* has been identified as the main causal agent of the disease for its high degree of virulence [6]. This hemibiotrophic basidiomycete causes lethal effects in plants by degrading the lignin and cellulose of the cell walls of the stem through the action of ligninolytic enzymes that affect the xylem, and consequently the transport of nutrients and water, thereby affecting biochemical and physiological processes that impair the normal development of the plant [7,8].

Several studies on plants reveal the importance of determining the plant-pathogen relationship by assessing the pathogenicity and virulence of pathogenic microorganisms [9]. For oil palm, different artificial inoculation methods have been adopted using *Ganoderma*: inoculating seedlings in vitro with mycelia produced in the medium to assess their interaction with the roots [10], inoculating seedlings in vitro at the base with mycelia [11], spraying the roots of oil palm seedlings with a mycelial suspension [12], immersing the seedling roots in a mycelial suspension [13], inoculating the primary roots of pre-germinated seeds with 3.3 cm^3^, 27 cm^3^, 216 cm^3^ rubber wood blocks colonized with dikaryotic mycelia of *Ganoderma* [14], and inoculating four-month-old seedlings [15] and six- to twelve-month-old plants [16] with rubber wood (*Hevea brasiliensis*) blocks colonized with dikaryotic mycelia of *Ganoderma* spp. [14].

The last method has been used to assess the pathogenic capacity of different species of *Ganoderma* [14], the importance of the potential importance of the inoculum within the pathosystem [17], the manifestation of symptoms [2], the aggressiveness of different isolates [18], the susceptibility of different oil palm varieties to the disease [19], the effectiveness of different biological controllers as an integrated disease management strategy [15,20,21], and the importance of nutrition as a defense mechanism of the plant against the pathogen [22,23,24].

Studies carried out in Colombia relate the symptoms found in plants affected by BSR with the presence of basidiocarps at their base and the extraction of isolates of *Ganoderma* spp [25]. However, this study did not determine the pathogenicity of isolates of *Ganoderma* spp. related to the disease. Therefore, to determine the pathogenic association of *Ganoderma* with BSR in northern Colombia, it is necessary to isolate basidiocarp of the pathogen from diseased oil palms, to test the pathogenicity of the different isolates, and to morphologically and molecularly identify the pathogenic microorganisms in oil palm.

## 2. Materials and Methods

### 2.1. Description of Symptoms and Preparation of Isolates of Ganoderma spp.

The external and internal symptoms of diseased oil palms were detected in plantations with reported cases of BSR in northern Colombia. Basidiocarp of *Ganoderma* were collected from those diseased oil palms and then transported to the Laboratory of Phytopathology of Cenipalma. Under laminar flow conditions, the surface of the basidiocarps was disinfected with 70% alcohol. These were then divided into two parts. One part was cut into small portions of approximately 5 mm in diameter and was disinfected with 70% alcohol, 1% hypochlorite, and sterile distilled water, and then seeded in Petri dishes with selective culture medium for *Ganoderma* (GSM) [26]. The other part of the basidiocarp was used to extract the context tissue and then was directly seeded in the culture medium GSM and Malt extract agar (MEA) [27]. The Petri dishes were incubated at 26 °C in the dark. The presence of the microorganism was tested four to seven days later, and the hyphae tips that grew out from the basidiocarp segments were sub-cultured and maintained into fresh plates containing MEA.

### 2.2. Preparation of Inoculum

The rubber wood (*Hevea brasiliensis*) blocks methodology described by [19,27,28] was used with some modifications to prepare the inoculum. Briefly, rubber wood blocks (6 cm × 6 cm × 6 cm) were oven dried at 70 °C for 24 h. Next, they were sterilized in an autoclave for 2 h at 121 °C and 15 psi. Once sterilized, they were immersed in a solution with 3% Malt extract for 24 h. They were then separated into individual blocks and sterilized again. Finally, each block was inoculated with ten discs taken from actively growing *Ganoderma* cultures and incubated at 27 °C in the dark for five months.

### 2.3. Assessment of the Pathogenicity of the Isolates

The test was established in the municipality of Aracataca-Magdalena (10°34′11.7″ N 74°11′7.33″ W) in the shade under a 50% shade cloth and in the local environmental conditions (mean temperature of 27 °C, relative humidity of 86%, and mean annual rainfall of 1545 mm). The plant material used in the study consisted of four-month-old *Elaeis guineensis* (Dura x Pisífera) oil palm nursery seedlings, which were transplanted into new bags with a sterilized substrate composed of soil and organic matter in a 4:1 ratio. At planting, a colonized rubber wood block was placed in direct contact with the root system.

A total of seven isolates were assessed via this test using a completely randomized design. The number of treatments corresponded to the number of isolates plus two controls (control with rubber wood block without pathogen and an absolute control without any rubber wood block). There were five replicates and the experimental unit corresponded to a total of ten plants. This test was carried out twice, once in 2018 and once in 2019.

The internal and external symptoms of the inoculated plants were registered. The pathogenicity of the isolates was assessed based on the external symptoms observed weekly till 29 weeks post-inoculation (wpi) using the disease severity scale designed by [29], the disease severity index (DSI) with values between 0 and 100 according to the formula proposed by [29], and the severity of foliar symptoms (SFS) of [30].

For seedlings showing both external and internal symptoms, fresh tissues with visible lesions were collected from the advance zone in the bulb and root, and then the surface was sterilized following the methodology described above. Thereafter, fragments of approximately 5 mm were seeded in an MEA culture medium. The isolate was purified seven days after seeding, and the morphology of the mycelia of *Ganoderma* spp. was observed under an optical light microscope (magnification 40×) and we observed the clamp connection structure of *Ganoderma*.

### 2.4. Assessment of Somatic Compatibility of the Isolates of Ganoderma spp.

To assess genetic similarity using the somatic compatibility test, seven isolates of *Ganoderma* spp. prepared from oil palms affected by BSR were tested on 2% MEA medium, placing a 0.5 mm plug for each isolate and pairing them in all combinations, using self-crosses as controls, as reported by [31]. Two plugs were placed per 9-mm Petri dish with culture medium at 2 cm. They were incubated in the dark at 25 °C for 14 days. Three Petri dishes were used per interaction and assessed at the end of the test using the antagonism scale [32].

### 2.5. Molecular Identification of Isolates Associated with BSR

Seven isolates were grown in a Malt extract liquid culture medium containing 15% Malt extract and 5% yeast extract, and then filtered. The mycelia were macerated with liquid nitrogen and stored at −70 °C until use. DNA was extracted following the standardized protocol of the Laboratory of Molecular Biology of Cenipalma, modified from [33]. Briefly, 100–200 mg of macerated tissue was mixed with 700 μL of extraction buffer (0.5 M NaCl, 0.2 M Tris pH 8.0, 10 mM EDTA pH 8.0, 1% SDS) preheated to 65 °C. After 2 h of incubation at 65 °C, the supernatant was collected from a centrifugation at 8000 rpm for 15 min and previously purified with an equal volume of phenol: chloroform: isoamyl alcohol (25:24:1), the supernatant was purified with chloroform: isoamyl alcohol (24:1) and centrifugated at 8000 rpm for 15 min, and the aqueous phase was collected. DNA was precipitated with cold isopropanol for at least 16 h at −20 °C. After centrifugation, the pellet was washed with 70% ethanol, dried and resuspended in an appropriate volume of 1× TE pH 8.0 according to the size of the pellet. The DNA was visualized on 1.0% agarose gels (1× TAE, 4 μL of DNA per well plus 2 μL of EZ-Vision (Amresco, Framingham, MA, USA) via electrophoresis for 30 min at 100 volts and was quantified via spectrophotometry using a Synergy MX (BioTek, Santa Clara, CA, USA).

Partial regions of *ITS*, *rpb2*, and *tef1-α* were amplified using the primers forward/reverse ITS1F/ITS4b [34], f*RPB2*-5F/b*RPB2*-7R2 [35,36], and EF1-983F/EF1-2218R [37], respectively. PCR was carried out in a 15 μL solution composed of 13 μL of master mix and 2 μL of DNA. The 13 μL of master mix consisted of 7.4 μL H_2_O, 1× of 10× PCR-buffer, 1.5 mM of MgCl2, 0.5 μM of each primer of three regions, 0.2 mM of dNTPMix, and 0.3 U Taq-polymerase. The amplification parameters for the *ITS*, *rpb2*, and *tef1-α* regions were as follows: initial denaturation at 95 °C for 3 min, 40 cycles of amplification with denaturation at 95 °C for 35 s, annealing for 35 s at 60 °C for *ITS* and at 55 °C for *tef1-α* or for 1 min at 52 °C for *rpb2*, and extension at 72 °C for 1 min, with a final extension of 10 min at 72 °C. The PCR products were visualized in 1.5% agarose gels (1× TAE, 4 μL of PCR product per well plus 2 μL of EZ-Vision (Amresco, Framingham, MA, USA) using electrophoresis for 45 min at 100 volts. The PCR products were purified and sequenced using Macrogen Inc. (Seoul, South Korea) with an automated ABI3700 DNA sequencer with the primers used in each PCR.

The sequencing results were edited, and the consensus sequences were constructed using Sequencher™ 5.3 (Gene Codes Corporation, Ann Arbor, MI, USA). Once the sequences had been edited, identity was confirmed against the GenBank database with the BLASTn algorithm (Basic Local Alignment Search Tool, http://www.ncbi.nlm.nih.gov/BLAST accessed on 20 October 2021). The sequences of this study along with 14 reference sequences (accession numbers in Table 1) were then aligned and edited using the MUSCLE algorithm in MEGA X [38]. The alignments of each region were concatenated in MEGA X, and the resulting gaps were inspected manually. With the final matrix of the three concatenated regions, the nucleotide substitution model based on the Bayesian information criterion (BIC) was determined using MEGA X for subsequent phylogenetic analysis. The phylogenetic analysis was carried out using the maximum likelihood (ML) method based on the T92+G model (Tamura 3 parameters using the gamma distribution) [33]. The support for nodes was carried out using the bootstrapping method with 1000 pseudoreplicates. The species *Tomophagus colossus* was included as a related external group (Table 1).

## 3. Results

### 3.1. Description of Symptoms and Preparation of Isolates

The oil palms affected in the field by BSR presented foliar symptoms, such as bending of the lower leaves, which can break at some point along the rachis forming a raceme, followed by drying and necrosis of the leaves. The newer leaves showed abnormal growth (shortening), and their leaflets were thinner and more fragile (leaflet bending) compared to those of healthy oil palms. There was also an accumulation of multiple spears that remained unopened, and the palms had a vase-like appearance in the middle-third of the leaves upwards as they were more erect than normal.

**Figure 1:**
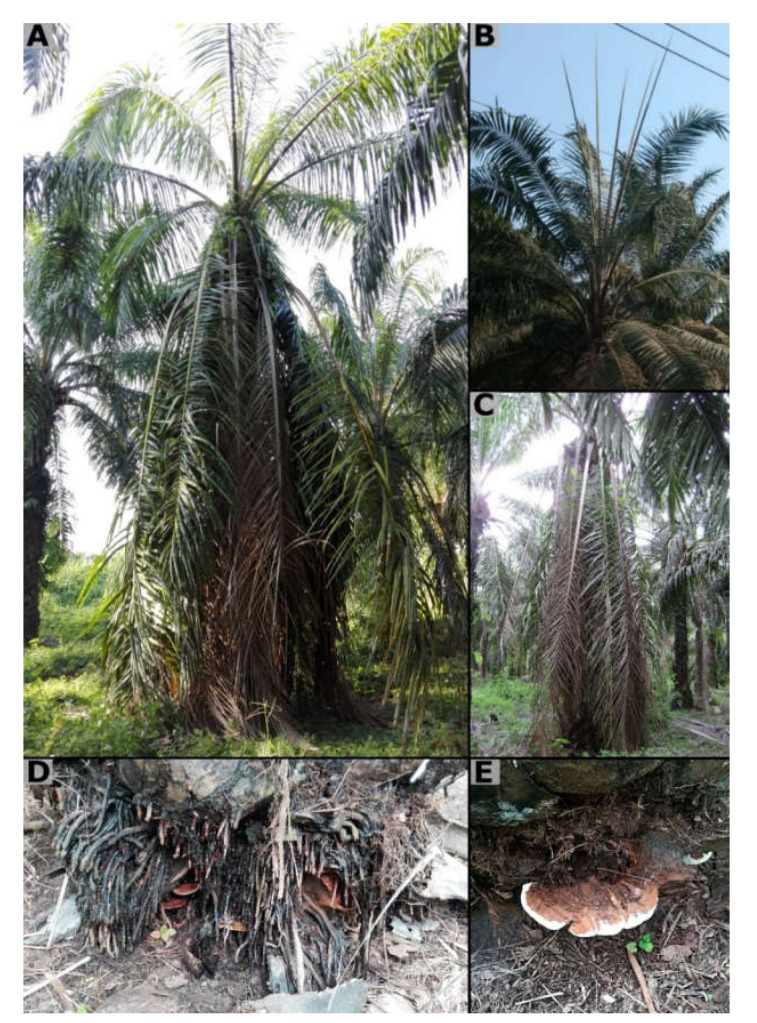
External symptoms observed in oil palms affected by BSR in the field in Colombia. (**A**) Oil palm with symptoms of wilting and chlorosis. (**B**) Unopened spears. (**C**) Snapping/flattening of the crown. (**D**) Formation of adventitious roots. (**E**) Typical basidiocarps of *Ganoderma zonatum* on a BSR affected palm.

In general, the affected oil palms showed thickening and accumulation of adventitious roots, which are friable, dry, and have a corky appearance. There were also fructifications of the pathogen in the form of small primordia (initially) or basidiocarps with a lacquered surface and whitish border at the base of the stem and in those roots (Figure 1). At the level of the stem, craters can occur in very advanced cases of the disease.

**Figure 2:**
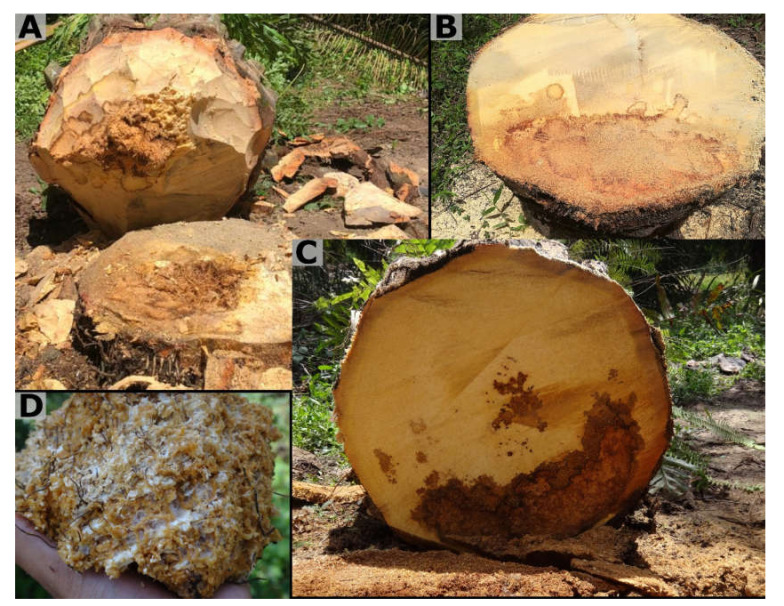
Internal development of lesions of *Ganoderma* in oil palms in Colombia. (**A**,**B**) Presence of lesions with irregular border (**C**) Delimitation of light and dark areas. (**D**) Presence of mycelium in debris stem.

An internal stem rot was observed, characterized by the appearance of areas with different colors or brown shades (from dark to light). These areas are furrowed by bands or lines of darker colors that give them the appearance of lesions on a map. Cross-sections of the stem showed bright yellow transitional areas between the affected and healthy tissues corresponding to disease progression areas (Figure 2). In adult oil palms, this rot develops more frequently laterally around the stem, that is, the rot goes from the outside to the inside, compared to the rot in young oil palms, which develops from the innermost part (center of the stem) to the outside.

### 3.2. Isolates and Pathogenicity Tests

Seven isolates of *Ganoderma* were obtained from oil palm plantations in northern Colombia, with the following Cenipalma isolate bank codes: CPBsZN-01-29, CPBsZN-02-30, CPBsZN-03-31, CPBsZN-04-34, CPBsZN-05-35, CPBsZN-06-36, and CPBsZN-07-38.

All isolates were pathogenic to oil palm when inoculated in seedlings.

**Figure 3:**
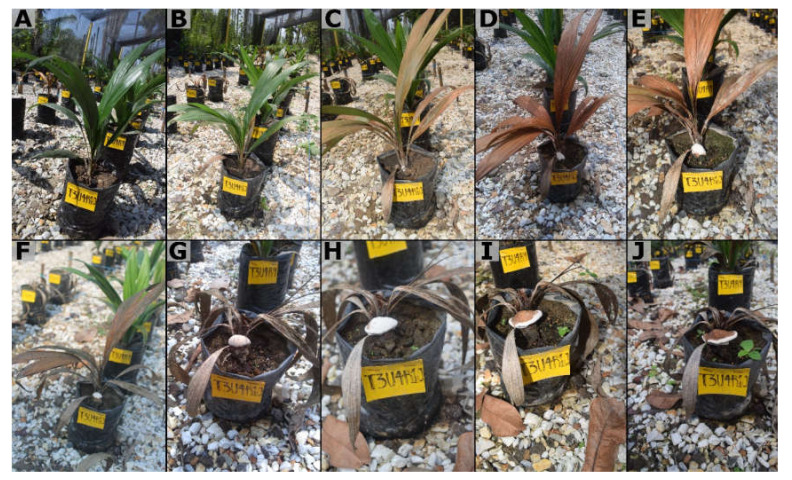
Progression of the symptom of basal stem rot in four-month-old oil palm seedlings inoculated with *Ganoderma zonatum.* (**A**) Asymptomatic infected seedlings at 15 weeks post inoculation (wpi). (**B**) Seedling infected at 18 (wpi) with necrosis on lower leaves. (**C**) Seedling at 21 wpi with generalized necrosis and mycelium formation at the base of the stem. (**D**–**F**) Infected seedling >23 wpi with visible basidiocarp. (**G**–**J**) seedling infected at >26 wpi shows complete basidiocarp formation.

**Figure 4:**
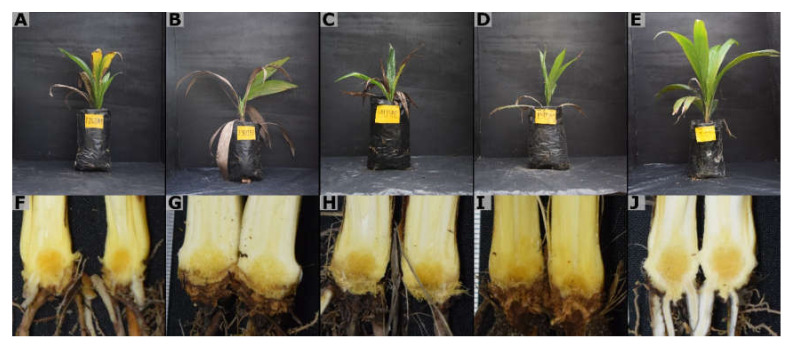
Oil palm seedlings exhibit external and internal symptoms. (**A**–**D**) Foliar symptoms in plants inoculated. (**F**–**I**) Bulb of the infected seedlings show brown coloration and necrosis. (**E**,**J**) Control (uninoculated) without signs of necrosis and healthy bulb.

The appearance of symptoms was observed from wpi 6. The external symptoms included a slight initial chlorosis, which quickly turned into a necrosis from the apex of the leaves and progressed to affect the entire leaf blade. Some plants showed the development of a mycelial mass at the base, which progressively advanced to a small white button that gave rise to a well-developed *Ganoderma* basidiocarp. At advanced stages of symptom development, the plants became completely necrotic and died, with or without the presence of the signs (Figure 3). The internal symptoms were characterized by necrosis of the primary roots and stem bulb, symptoms not observed in the non-inoculated controls (Figure 4).

**Figure 5:**
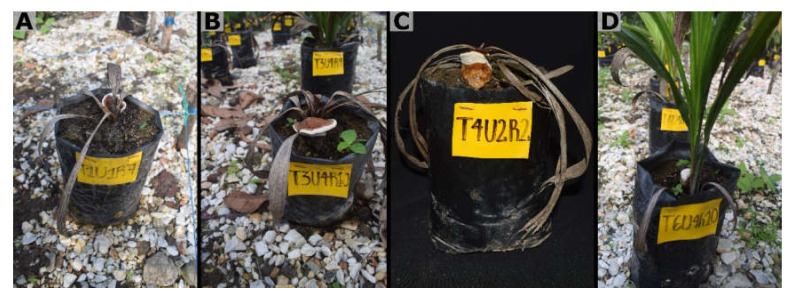
Seedlings inoculated with isolates of *Ganoderma zonatum* that presented the formation of basidiocarp. (**A**) CPBsZN-01-29. (**B**) CPBsZN-03-31. (**C**) CPBsZN-04-34. (**D**) CPBsZN-06-36.

Of the seven isolates of *Ganoderma*, only four (CPBsZN-01-29, CPBsZN-03-31, CPBsZN-04-34, and CPBsZN-06-36) showed signs of the pathogen, along with root and bulb necrosis. The presence of *Ganoderma* was confirmed after growth in GSM medium and is characterized by white mycelial growth and the presence of brown halos around the colony. The non-inoculated plants (negative control) showed no signs (Figure 5).

The appearance of signs occurred from wpi 12 in seedlings inoculated with isolate CPBsZN-03-31, with the development of a white mycelial mass; however, such mycelial development started at wpi 16, 18, and 21 for isolates CPBsZN-06-36, CPBsZN-01-29, and CPBsZN-04-34, respectively.

The incidence of BSR in inoculated seedlings in the first weeks (wpi 12 to 16) was 9% for isolate CPBsZN-05-35, 10% for isolates CPBsZN-04-34 and CPBsZN-06-36, and 12% for isolate CPBsZN-03-31. From wpi 17 to 20, the incidence ranged from 52 to 82%, with isolate CPBsZN-07-38 reaching the highest percentage. The severity index during this period ranged from 11 to 19.33% for isolates CPBsZN-01-29 and CPBsZN-06-36, with significant differences only for the latter. At the end of the assessments at wpi 27 to 29, the highest incidence of 100% was found for seedlings inoculated with isolate CPBsZN-06-36, with a severity index of 34%, and the lowest incidence was 94% for isolate CPBsZN-02-30, with a severity index of 23.5% (Table 2).

**Figure 6:**
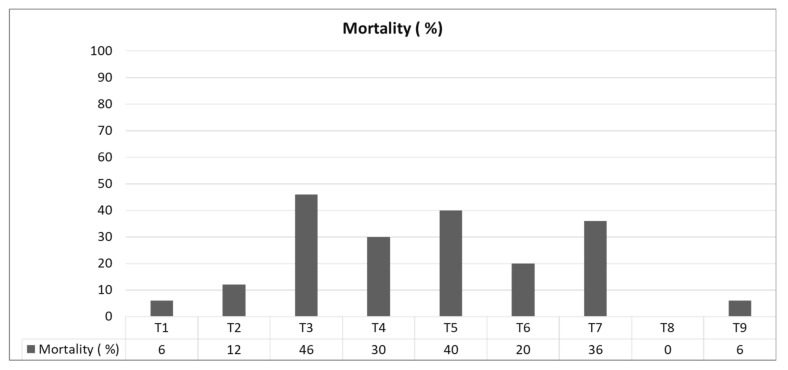
Mortality rate in nursery oil palms inoculated with isolates of *Ganoderma* obtained from oil palms affected by BSR in northern Colombia after 29 weeks.

The SFS at wpi 12 was 71.98 and 70.80% for isolates CPBsZN-05-35 and CPBsZN-07-38, respectively, followed by isolate CPBsZN-03-31 with a SFS of 70.26%. At wpi 29, isolates CPBsZN-03-31 and CPBsZN-05-35 reached a SFS of 73.22 and 73.25%, respectively, while isolate CPBsZN-04-34, which showed the highest number of basidiocarps (6 seedlings), reached a SFS of 64.7%. Isolates CPBsZN-01-29, CPBsZN-03-31, and CPBsZN-06-36 each had four seedlings with basidiocarps, with a SFS of 56.54, 59.96, and 69.34%, respectively. As for the bulb severity index (BSI), all isolates of *Ganoderma* had some degree of necrosis compared to controls, with isolates CPBsZN-03-31, CPBsZN-05-35, and CPBsZN-07-38 showing the highest severity with percentages of 49, 45, and 37%, respectively. Although there were no basidiocarp in some treatments, the bulbs were affected by the pathogen. Likewise, these isolates showed the highest mortality rate, with values of 46, 40, and 36%, respectively (Figure 6). The pathogenicity was confirmed by the re-isolation of *Ganoderma* from the bulbs of inoculated seedlings.

### 3.3. Assessment of Somatic Compatibility of Isolates of Ganoderma

**Figure 7:**
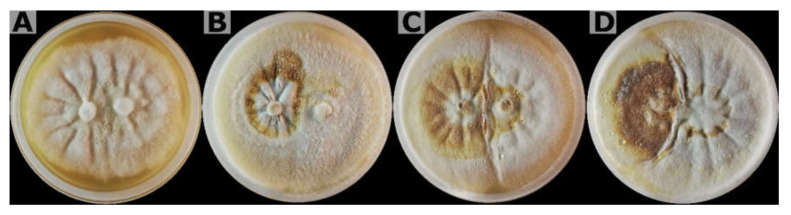
Somatic compatibility of seven isolates of *Ganoderma* using in this study, showing formation of inhibition zone or barrage in incompatible reactions. (**A**) compatible reaction of self-pairing (control) between CPBsZN-01-29; (**B**) incompatible reaction between isolates CPBsZN-03-31 and CPBsZN-07-38 (weak interaction); (**C**) incompatible reaction between isolates CPBsZN-04-34 and CPBsZN-05-35 (moderate interaction); and (**D**) incompatible reaction between isolates CPBsZN-02-30 and CPBsZN-03-31 (strong interaction).

All possible pairings of the assessed strains presented incompatibility since an inhibition zone or barrier line was formed and presented different levels of incompatibility (weak, moderate, and strong), except for the self-crosses (control) in which the colonies merged into one (Figure 7). These results indicate that the isolates are genetically different and originate from a different inoculum.

### 3.4. Molecular Identification of Isolates Associated with BSR

**Figure 8:**
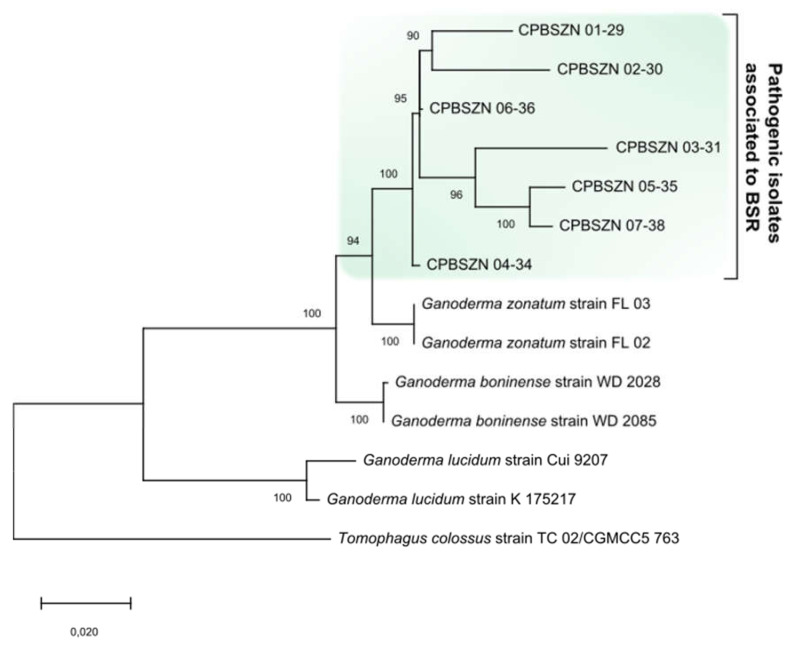
Phylogenetic tree of isolates associated with BSR based on data provided by a combination of the *ITS*, *rpb2*, and tef1α regions. The numbers above the nodes represent bootstrap values. The topology and values are derived from maximum likelihood analysis.

Twenty-one sequences were generated in this study and deposited in GenBank: seven from *ITS* (MZ170061–MZ170068), seven from *tef1-α* (MZ197864–MZ197870), and seven from *rpb2* (MZ229332–MZ229338). The partial sequencing of the *ITS*, *rpb2* and *tef1-α* regions led to the identification, with a sequence identity greater than 97%, 98%, and 97%, of *Ganoderma zonatum* Murrill, 1902*,* respectively, compared with the references sequences of GenBank. Additionally, identities of 92.3–99.9% for *ITS*, 98–100% for *rpb2,* and 97–100% for *tef1-α* were found among the sequences in this study. The alignment based on the combination of the three regions comprised a set of 14 taxa with a total consensus of 2116 characters, including gaps (722 from *ITS*, 914 from *rpb2*, and 453 from *tef1-α*), corresponding to 1310 conserved sites, 719 variable sites, 579 parsimoniously informative sites, and 212 singletons. Phylogenetic analysis grouped all isolates associated with BSR, identified with the name of *G. zonatum*, in a subclade of the reference sequences of *G. zonatum* with a support of 94%. Likewise, within the major clade, the species *Ganoderma boninense* was grouped with them, with a support of 100% (Figure 8).

## 4. Discussion

Our study established the pathogenic association and identification of isolates with BSR of oil palm in Colombia by assessing the incidence and severity index in semi-controlled conditions of infection, progression, and development of the disease, and the molecular proximity with the species *G. zonatum.* Moreover, the in vitro somatic incompatibility between isolates revealed genetic heterogeneity, thereby indicating, as suggested by [39], the importance of basidiospores in the infection cycle of this disease.

The symptoms associated with BSR caused by *G. boninense* in oil palm are deterioration in water absorption and nutrient deficiency in the foliage of the affected palms, chlorosis, the accumulation of spears, the bending of the leaves until flattening of the crown at advanced stages, and the presence of basidiocarps at the base of the plants [28,40,41,42], corroborating the symptoms observed in plantations affected by BSR in Colombia.

All the isolates obtained in our study corresponded to *G. zonatum* and were pathogenic in oil palm with different degrees of severity. Therefore, the presence of *G. boninense*, a species recognized as the main causal agent of BSR in producing countries such as Malaysia and Indonesia, has not yet been confirmed in Colombia [23,43,44,45]. This is supported by the findings of [4], who indicated that the dominance of *Ganoderma* species associated with BSR could vary depending on the location. Although *G. zonatum* showed a higher severity than *G. boninense* in artificial inoculations, the disease has continued to be restricted to two palm areas in Colombia with a relatively low incidence since its first records in 1994 [25,46,47].

Pathogenicity results showed that the isolates obtained in this study varied and were statistically different, corroborating the different aggressiveness among *Ganoderma* strains reported in numerous studies [5,14,48,49]. This is mainly due to genetic variations, inoculum potential, aggressiveness of the isolates and their interaction with external factors, such as temperature, humidity and solar radiation. [19]. The appearance of symptoms in the most aggressive isolate of our study was from wpi 12, corroborating the reports of [29] for inoculations with *G. boninense*. With regard to incidence, authors such as [17,50] report for *G. boninense* and [48] for *G. boninense* and *G. zonatum,* infection rates ranging from 60 to 100% at six and eight months post inoculation, agreeing with the results of this study, in which all isolates showed values above 90% at the end of the experiment.

Severity indices in the last assessment of the experiment ranged from 23 to 34%, and was lower than those reported by [29] and [51] who used the same disease severity scale in seedlings artificially inoculated with *G. boninense*, with values of 87 and 90.4%, respectively. However, our severity values are closer to those reported by [48] for *G. zonatum*, with values of 34.08 and 43.78% for two isolates analyzed in the study. Lastly, the mortality rates found in our study were higher than those reported for *G. zonatum* by [48].

Some authors, such as [17,28,52], reported that the initial post-inoculation symptoms by *Ganoderma* were foliar chlorosis followed by necrosis and drying; however, the first symptom observed in this study was necrosis from the apex to the base of the leaf. This initial symptom was similar to that reported by [53].

The genetic heterogeneity of the isolates agrees with numerous studies on *Ganoderma* [31,39,49,54,55,56], which indicated that intra- and interspecific somatic compatibility is not frequent in this genus. Therefore, it is suggested that the mode of dispersal between oil palms in northern Colombia is possibly based on basidiospores.

This study assessed three genomic regions for the genetic identification of the seven isolates which were associated with the species *G. zonatum*. One of these regions was the internal transcribed spacer that has been particularly useful in the identification of fungi [57], and is considered a rapidly evolving region [58,59]. In this study, the *ITS* region had a particular behavior and was observed, in data not shown, which the alignment based only of *ITS* region which comprised a set of nine taxa, seven sequences obtained in this study and two *G. zonatum* sequences from GenBank, with a total consensus of 722 characters, including gaps, corresponded to 623/722 conserved sites and 86/722 variable sites, additionally the results obtained of identities (92–98%), were made necessary to confirm the identity using other markers. The other two regions: second largest subunit of RNA polymerase II (*rpb2*) and translation elongation factor 1-α (tef1α) have been instrumental to resolve the ambiguous evolutionary relationships that exist among species of *Ganoderma* [35,36]. The *rpb2* region tends to resolve the main clades at high and low taxonomic levels compared to the tef1α region, which is strongly conserved [36]. Therefore, the combination of these three regions was able to discern conserved and variable regions among species of *Ganoderma*, thereby supporting the approximation of the species extracted in the isolates associated with BSR to *G. zonatum* in this study.

The phylogenetic approximation based on the *ITS*, *rpb2*, and *tef1-α* regions resulted in the close relation identification of *G. zonatum* with the isolates associated with oil palm BSR in this study, and this is the first report of the species in Colombia. All isolates from this region of Colombia were grouped in a single subclade belonging to clade C of the isolates of *G. zonatum* from Florida and *G. boninense* from Japan reported by [60], with the entire phylogenetic topology agreeing with that of the aforementioned study. However, the findings indicate the need for further studies to compare more molecular markers and more isolates of *G. zonatum*, including those from this study and those from other studies reported in Africa, Asia, the US, Argentina, and Brazil [60,61,62,63,64,65] to determine if the species has a different genetic diversity behavior and spatial distribution in different continents.

Finally, to our knowledge, this is the first report on the extraction, identification, and characterization of *G. zonatum* as a causal agent of BSR in Colombia. These results contribute to the knowledge of the biology of this pathogen in Colombia, and with the identification of the causal agent of BSR in Colombia, could lead to studies of genetic improvement, epidemiology, and management strategies of BSR that will allow for the minimizing of the incidence and spread of this disease in oil palm plantations in our country.

## Figures and Tables

**Table 1:** Information on the species used in the phylogenetic analyses.

Species	Voucher	Origin	GenBank Accession Number		
			*ITS*	*rpb2*	*tef1-α*
*Ganoderma boninense*	WD 2028	Japan	KJ143905	KJ143964	KJ143924
*Ganoderma boninense*	WD 2085	Japan	KJ143906	KJ143965	KJ143925
*Ganoderma zonatum*	FL 02	FL-USA	KJ143921	KJ143979	KJ143941
*Ganoderma zonatum*	FL 03	FL-USA	KJ143922	KJ143980	KJ143942
*Ganoderma lucidum*	Cui 9207	Yunnan, China	KJ143910	KJ143970	KJ143928
*Ganoderma lucidum*	K 175217	UK	KJ143911	KJ143971	KJ143929
*Tomophagus colossus*	TC 02	Vietnam	KJ143923	-	KJ143943
*Tomophagus colossus*	CGMCC5 763	Philippines	-	JQ081070	-

**Table 2:** Severity index in plants inoculated with *Ganoderma zonatum*.

	Isolate	Disease Incidence (Weeks Post Inoculation) (%)				Mean Disease Severity Index (DSI) (Weeks Post Inoculation) (%)			
		20	23	26	29	20	23	26	29
T1	CPBsZN-01-29	52	88	90	96	11.00 (2.8) c	20.67 (3.54) b	26.50 (1.73) bc	28.83 (0.57) c
T2	CPBsZN-02-30	68	88	88	94	15.16 (1.6) b	21.17 (1.60) b	22.50 (0.50) d	23.50 (0.50) e
T3	CPBsZN-03-31	66	90	98	98	15.00 (1.3) b	23.00 (1.32) b	28.00 (2.00) b	30.67 (0.28) b
T4	CPBsZN-04-34	68	86	92	96	15.33 (2.5) b	22.33 (1.89) b	26.67 (1.52) bc	31.16 (1.60) b
T5	CPBsZN-05-35	70	96	98	98	16.50 (2.2) ab	22.33 (2.25) b	25.00 (0.00) c	24.67 (0.28) d
T6	CPBsZN-06-36	74	92	98	100	19.33 (2.3) a	27.50 (1.00) a	30.67 (1.60) a	34.00 (0.50) a
T7	CPBsZN-07-38	82	92	98	98	18.33 (2.0) ab	23.17 (0.28) b	24.67 (0.28) c	24.50 (0.00) de
T8	Control 1	0	0	0	0	0.00 d	0.00 c	0.00 e	0.00 f
T9	Control 2	0	0	0	0	0.00 d	0.00 c	0.00 e	0.00 f

DSI means followed by the same letter are not significantly different with a *p* = 0.05 according to Duncan’s multiple range test. Values within parentheses correspond to the standard deviation.

## Data Availability

The data presented in this study are available on request from the corresponding author.
